# Association between intraoperative noise intensity during hip arthroplasty and the postoperative risk of kinesiophobia: a prospective cohort study

**DOI:** 10.3389/fsurg.2026.1945025

**Published:** 2026-09-10

**Authors:** Jiang Jian, Aijun Xu, Yaoling Dai, Zheyu Huang, Long Zhang, Jiang Li

**Affiliations:** 1 Department of Operation room, Ningbo No.6 Hospital, Ningbo, Zhejiang, China; 2 Ningbo Clinical Research Center for Orthopedics, Sports Medicine & Rehabilitation, Ningbo, Zhejiang, China

**Keywords:** correlation, hip arthroplasty, intraoperative noise, kinesiophobia, pain assessment, predictive model

## Abstract

**Objective:**

The study aimed to investigate the association between intraoperative noise during hip arthroplasty and the development of postoperative kinesiophobia, as well as the predictive value of intraoperative noise for postoperative kinesiophobia, and associations of intraoperative noise with short-term postoperative pain, anxiety, and early functional recovery.

**Methods:**

This prospective cohort study enrolled patients undergoing unilateral primary total hip arthroplasty under spinal anesthesia. Patients were divided into a high-noise group (> 70 dB) and a low-noise group (≤ 70 dB) according to the level of intraoperative ambient noise. The primary outcome was the Tampa Scale of Kinesiophobia (TSK-17) score on the third postoperative day. Secondary outcomes included Visual Analog Scale (VAS) scores (days 1 and 3), Hospital Anxiety and Depression Scale (HADS) scores (day 1), and Harris Hip Score (HHS) (day 3). The association between noise level and TSK score was assessed using Spearman correlations, while multivariate logistic regression evaluated intraoperative noise as a predictor of Kinesiophobia, and independent associations between other postoperative variables and Kinesiophobia were investigated. The discriminative accuracy of intraoperative noise for predicting Kinesiophobia was examined using receiver operating characteristic (ROC) curves.

**Results:**

TSK scores were significantly higher in the high-noise group than in the low-noise group (43.2 ± 5.6 vs. 35.8 ± 5.0, *P* < 0.001). VAS scores were also significantly higher on postoperative day 1 (5.4 ± 1.3 vs. 4.0 ± 1.1) and day 3 (5.1 ± 1.2 vs. 3.6 ± 1.0; both *P* < 0.001). HADS scores were higher (10.8 ± 3.0 vs. 8.9 ± 2.4, *P* < 0.001) in the high-noise group, while HHS scores were lower (69.5 ± 7.4 vs. 76.2 ± 6.8, *P* < 0.001). Intraoperative noise level correlated positively with the TSK score (*r* = 0.600, *P* < 0.001) and was also independently predictive of postoperative kinesiophobia (OR=1.18, 95% CI: 1.09–1.29, *P* < 0.001). ROC curves showed intraoperative noise predicted kinesiophobia with an AUC = 0.814; optimal cutoff = 72.3 dB (sensitivity = 82.1%, specificity = 75.4%). Lastly, VAS and HADS scores on postoperative day 3 were both independently associated with kinesiophobia (both *P* < 0.01).

**Conclusion:**

Higher levels of intraoperative noise during hip arthroplasty were significantly associated with short-term kinesiophobia, increased pain perception, fear of movement, and reduced early functional recovery.

**Clinical trial registration:**

www.medicalresearch.org.cn, identifier MR-33-26-021584.

## Introduction

Total hip arthroplasty (THA) is an effective surgical procedure for treating end-stage hip-joint disorders, and is widely used in patients suffering from osteoarthritis, rheumatoid arthritis, femoral head necrosis, and severe trauma. It has demonstrated remarkable clinical efficacy in improving joint function and relieving pain (1). The volume of THA surgeries is increasing steadily with the aging of the population and increased patient expectations for quality of life. In addition, enhanced postoperative rehabilitation and patient satisfaction have increasingly become focal points of research (2).

Studies have indicated that early postoperative rehabilitation is affected not only by physiological factors but is also significantly influenced by psychological issues (3, 4). Of these, kinesiophobia has attracted extensive attention from both clinicians and researchers. Kinesiophobia refers to a persistent, irrational fear of movement or activity caused by a fear of injury or re-injury (5). It has been found that the development of kinesiophobia after THA can directly reduce patients’ willingness to undertake exercises for the restoration of function, thereby delaying recovery of joint function and potentially leading to long-term restrictions in joint mobility and reduced quality of life (6, 7). Therefore, early identification of patients at increased risk of developing postoperative kinesiophobia followed by targeted intervention is important for improving the overall prognosis of THA.

Most studies to date have focused on associations between postoperative kinesiophobia and preoperative psychological status, pain sensitivity, and other patient-related factors, with limited investigation of the potential effects of factors linked to the intraoperative environment (8, 9). Noise is one of the most common environmental pollutants in the operating room. Recent monitoring data from multiple studies have shown that the average noise level in orthopedic operating rooms far exceeds the limit of 40 dB stipulated by the World Health Organization, and peak intensities above 70 dB or even 90 dB are not uncommon during surgery (10). This high level of noise in the operating room not only affects the communication and concentration of the medical staff, but has also been reported to be associated with increased anxiety and blood pressure fluctuation in awake patients during the surgery (11). To date, most research on intraoperative noise has focused on the physical and mental stress experienced by medical staff, and the impact of intraoperative noise on the postoperative prognosis of awake patients under regional anesthesia is poorly understood (12). Excessive perioperative noise causes adverse psychological and physiological impacts on surgical patients. It triggers stress responses and worsens postoperative pain and recovery even under general anaesthesia. Specifically, the duration of exposure to noise levels exceeding 70 decibels independently correlates with aggravated postoperative pain intensity. Noise-cancelling headphones and other noise isolation measures are effective non-pharmacological interventions for improvement. Implementation of noise-reduction strategies during surgical procedures may significantly reduce the incidence of postoperative delirium, alleviate postoperative pain, and lower postoperative analgesic consumption. Additionally, such interventions attenuate perioperative psychological stress and lower postoperative stress marker level.

Total hip arthroplasty is frequently performed under spinal anesthesia (13). In this mode, patients maintain a clear state of consciousness throughout the operation and can clearly perceive sounds and environmental stimuli in the operating room (14, 15). This suggests that under these conditions, intraoperative noise may have a more sustained impact on the patient’s psychological state compared with general anesthesia, and may conceivably affect postoperative psychological perceptions and motivation for rehabilitation (14, 16).

This study adopted a prospective cohort design to explore the association between the intensity of intraoperative noise and short-term postoperative prognosis of patients undergoing THA under spinal anesthesia. The study focused on analyzing the predictive effect of intraoperative noise on postoperative kinesiophobia and clarifying its relationship with postoperative pain, anxiety, and early functional recovery, to provide a reliable reference for improving the perioperative environmental management of THA and enhancing postoperative rehabilitation.

## Methods

### Patients

This prospective cohort study recruited patients who underwent primary unilateral THA at a large orthopedic center between November 13, 2025, and February 22, 2026. All study participants received comprehensive information on the details of the study before enrollment and provided written informed consent. The protocol was approved by the Ethics Committee of Ningbo No. 6 Hospital (Approval No.: 2025-17 K) and has been registered with the China Medical Research Registration System under registration number MR-33-26-021584.

The inclusion criteria were as follows: patients aged between 18 and 80 years (without sex restriction); normal preoperative cognitive function and no impaired communication ability; voluntary participation with provision of written informed consent. The proposed method of anesthesia was spinal anesthesia. The exclusion criteria were as follows: patients who underwent other major surgeries during the same period; the presence of severe underlying diseases; individuals with impaired hearing, mental disorders, or those unable to accurately complete the scale assessments. The elimination criteria included the development of severe intraoperative complications or the need for transfer to the intensive care unit (ICU), missing intraoperative recording data, or refusal to continue participation in the study.

All surgeries were performed by an experienced and relatively stable joint surgery team. A standardized Zimmer artificial hip prosthesis (Versys series, Zimmer Biomet, USA) was utilized during the procedures, as were standardized surgical instruments, such as the Stryker high-frequency electric drill, electric oscillating saw, and bone hammer.

### Sample size

A two-sided test was used to calculate the required sample size based on pre-experimental data showing postoperative kinesiophobia incidence rates of 48% in the high-noise group and 22% in the low-noise group, with *α* = 0.05 and power of 1-*β* = 0.8. The minimum overall sample size was calculated as 94 cases using PASS 15.0 software. Allowing for a 10% loss-to-follow-up rate, an enrollment of 104 patients was ultimately planned, and 104 eligible patients were subsequently enrolled and grouped according to the predetermined intraoperative noise levels, resulting in a low-noise group (mean decibel value ≤ 70 dB, *n* = 52) and a high-noise group (mean decibel value > 70 dB, *n* = 52).

### Randomization and blinding

All enrolled patients were classified into a low-noise group (average intraoperative noise ≤70 dB) or a high-noise group (average intraoperative noise >70 dB) based on the mean sound level recorded during the entire surgical procedure. This classification was performed retrospectively by researchers who were not involved in patient care or outcome assessment, using audio-monitoring data collected during surgery. To minimize assessment bias, the postoperative evaluators were blinded to the group assignments of the patients. However, blinding of the surgical team was not feasible due to the observational nature of the study, as surgeons and anesthesiologists were inherently aware of the noise conditions in their operating rooms.

### Anesthesia and surgery

A standard intravenous line was established and connected to an ECG monitoring device to enable continuous monitoring of the patient’s heart rate, non-invasive blood pressure, and oxygen saturation. For the subarachnoid block anesthesia, an experienced senior anesthesiologist performed a lumbar puncture to confirm correct insertion of the needle into the subarachnoid space with unobstructed flow of the cerebrospinal fluid, after which 0.5% ropivacaine was administered at a constant rate. After completion of the injection, the patient’s position was adjusted to maintain the anesthesia level at T10. No additional sedatives were administered during the surgery to ensure that the patient remained conscious throughout the procedure. All patients had identical standardized perioperative analgesia protocols to avoid interference from different pain management strategies on postoperative pain assessment.

The surgery was performed as a unilateral primary THA by the orthopedic fixation team in accordance with a standardized protocol. The key steps involved positioning of the patient, disinfection with draping, establishment of surgical access, resection of the femoral head, preparation of the acetabulum and insertion of the prosthesis, preparation of the femoral canal, and placement of the prosthesis, trial reduction and assessment of stability, layered suturing, and fixation of the dressing. The stages of acetabular filing, bone surface preparation, and insertion of the prosthesis involved the utilization of instruments, including electric saws, high-frequency drills, and impact hammers, which were associated with high-intensity noise; these stages constituted the periods of highest intraoperative noise exposure. To ensure the comparability of noise-exposure records among patients, noise monitoring encompassed the entire surgical procedure from the incision to the closure of the suture.

Upon the completion of surgery, all patients were transferred to the post-anesthesia care unit for observation. They were returned to the general wards when their vital signs had stabilized.

### Noise monitoring

During the procedure, a high-precision integral sound level meter (model 831C; Larson Davis, USA) was used for the acquisition of acoustic data. The device was fixed approximately 1.2 m anterior to the patient’s head to minimize interference with surgical operations while maintaining proximity to the patient’s perceived sound field. The sampling frequency was set at 1 Hz, with recording of the average sound pressure level (Leq), peak sound pressure level (Lpeak), and maximum sound pressure level (Lmax) throughout the surgical process. The system automatically calculated the average level of intraoperative noise in decibels, which was used as the basis for patient grouping.

### Outcomes

Baseline data were collected preoperatively, including age, sex, body mass index (BMI), history of underlying diseases, preoperative Harris Hip Score (HHS), and operative duration. During the preoperative preparation phase, all intraoperative noise data were uniformly recorded by researchers in charge of setting up the equipment. On postoperative days 1 and 3, assessments of patient pain and anxiety levels and dyskinesia scores were made by two research nurses. All scale evaluations were conducted in separate quiet rooms.

The primary outcome was the Tampa Scale for Kinesiophobia (TSK-17) score, which was used to assess the patient’s level of kinesiophobia on the third postoperative day. This scale comprises 17 items, with each rated on a 4-point scale, with a total score ranging from 17 to 68 points. Patients scoring above 37 points were classified as having high levels of tension. Items 4, 8, 12, and 16 are reverse-scored items. The Chinese version of the scale has undergone validity and reliability validation, with a Cronbach’s *α* coefficient of 0.778, indicating good internal consistency (17).

On the first and third postoperative days, patient pain intensity was assessed using the Visual Analog Scale (VAS), a linear scale ranging from 0 to 10, where 0 indicates no pain, and 10 indicates severe pain. Patients self-rated their pain levels according to postoperative subjective perception. Patients with VAS scores exceeding 5 were classified as having moderate to severe pain for subsequent analysis and statistical evaluation.

On the third postoperative day, the early functional status of the patients was assessed using the HHS. This scoring system comprises four dimensions: pain, daily function, deformity, and range of motion, with a total score of 100 points. A score ≥90 is classified as excellent, 80–89 as good, 70–79 as fair, and <70 as poor, thereby providing a reflection of the patient’s short-term postoperative functional recovery (18).

On the first and third postoperative days, the anxiety levels of the patients were assessed using the Hospital Anxiety and Depression Scale (HADS). The scale consists of an anxiety subscale (HADS-A) and a depression subscale (HADS-D), each of which contains seven items and is scored on a 4-point scale ranging from 0 to 3, with total scores ranging from 0 to 21. Higher scores indicate higher levels of emotional distress. In this study, only the HADS-A scores were used for assessing anxiety, with scores ≥8 indicating the presence of anxiety symptoms. The Chinese version of this scale has been thoroughly validated for reliability and validity, making it suitable for assessing emotional states in Chinese patients (19).

### Statistical analysis

Data were analyzed using SPSS 26.0. Continuous variables are reported as mean ± standard deviation (x¯ ± SD) if normally distributed, and as median and interquartile range (median, IQR) if non-normally distributed. Categorical variables are presented as frequency and percentage (n, %). For intergroup comparisons, appropriate statistical methods were selected according to the data type and distribution, including the independent samples t-test, Welch’s corrected t-test, Mann–Whitney U test, or chi-square test (*χ*² test). Associations between variables were assessed using Pearson correlation coefficients. Factors influencing exercise phobia (defined by a Tampa Scale of Exercise Phobia score of TSK ≥ 37) were examined using a multivariate logistic regression model, with the results reported as odds ratios (OR) and 95% confidence intervals (95% CI). Additionally, the performance of the regression model was comprehensively evaluated, with assessment of model calibration using the Hosmer-Lemeshow test, calculation of the Nagelkerke R² to reflect the explanatory power of the model, evaluation of the model’s predictive accuracy using a contingency table, and the construction of a receiver operating characteristic (ROC) curve for analysis of the model’s discriminative efficacy. All statistical tests were two-sided, and *P* < 0.05 was defined as statistically significant.

## Results

**Figure 1:**
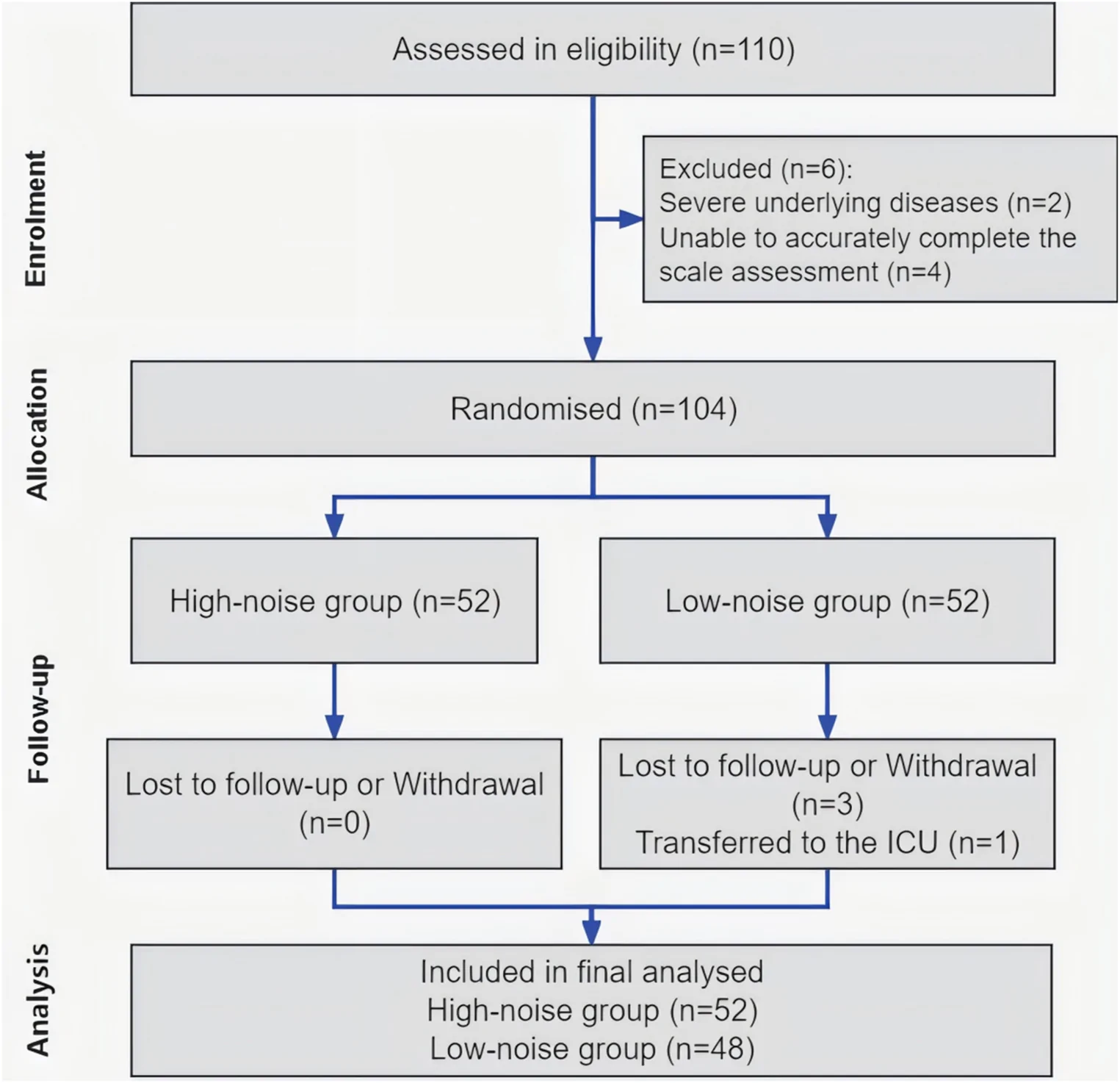
Flowchart of patient enrollment.

A total of 104 patients who underwent THA were enrolled in the study (Figure 1). One patient in the low-noise group experienced severe intraoperative complications and was subsequently transferred to the ICU; additionally, three patients were lost to follow-up or voluntarily withdrew from the study, resulting in the ultimate inclusion of 100 patients in the analysis, with 52 cases in the high-noise group and 48 in the low-noise group. There were no statistically significant differences between the two groups in terms of baseline characteristics, such as age, sex, BMI, preoperative HHS, operative duration, and history of underlying diseases (*P* *>* 0.05, Table 1).

**Table 1:** Baseline characteristics of patients in the two groups.

Indicators	High-noise group (*n* = 52)	Low-noise Group (*n* = 48)	*t*/*χ²*	*P*
Age (years), x¯ ± s	64.5 ± 6.9	63.9 ± 6.4	0.472	0.638
Sex (male/female)	25/27	23/25	0.016	0.899
BMI (kg/m²), x¯ ± s	24.3 ± 2.8	23.7 ± 2.5	1.139	0.258
Preoperative HHS, x¯ ± s	46.2 ± 8.1	47.0 ± 7.6	−0.509	0.612
Hypertension, *n* (%)	18 (34.6)	15 (31.3)	0.118	0.731
Diabetes, *n* (%)	9 (17.3)	7 (14.6)	0.133	0.716
Operative duration (min), x¯ ± s	82.6 ± 11.3	80.9 ± 10.7	0.781	0.437

BMI, body mass index; HHS, harris hip score; x¯, mean; S, standard deviation.

The operative duration and average intraoperative noise level were significantly higher in the high-noise group than in the low-noise group (*P* < 0.001). On postoperative days 1 and 3, the VAS scores in the high-noise group were significantly higher than those in the low-noise group (*P* < 0.001), while anxiety scores on day 1 also showed a marked increase (*P* < 0.001). On postoperative day 3, the TSK score was significantly elevated in the high-noise group (43.2 ± 5.6 vs. 35.8 ± 5.0, *P* < 0.001), while the HHS was significantly reduced (69.519 ± 7.406 vs. 76.188 ± 6.823, *P* < 0.001) (Table 2).

**Table 2:** Comparison of postoperative pain, anxiety, and hip joint function scores between the two groups.

Outcomes	High-noise group (*n* = 52)	Low-noise Group (*n* = 48)	*t*	*P*
Intraoperative average noise (dB), x¯ ± s	76.3 ± 4.2	65.8 ± 3.7	12.703	<0.001
T1 VAS score, x¯ ± s	5.4 ± 1.3	4.0 ± 1.1	6.054	<0.001
T2 VAS score, x¯ ± s	5.1 ± 1.2	3.6 ± 1.0	6.469	<0.001
T1 HADS score, x¯ ± s	10.8 ± 3.0	8.9 ± 2.4	3.695	<0.001
T2 TSK score, x¯ ± s	43.2 ± 5.6	35.8 ± 5.0	7.620	<0.001
T2 HHS score, x¯ ± s	69.5 ± 7.4	76.2 ± 6.8	−4.908	<0.001

T1, The first postoperative day; T2, The third postoperative day; VAS, visual analog scale; HADS, hospital anxiety and depression scale; TSK, tampa scale for kinesiophobia; HHS, harris hip score, x¯, mean; S, standard deviation.

**Figure 2:**
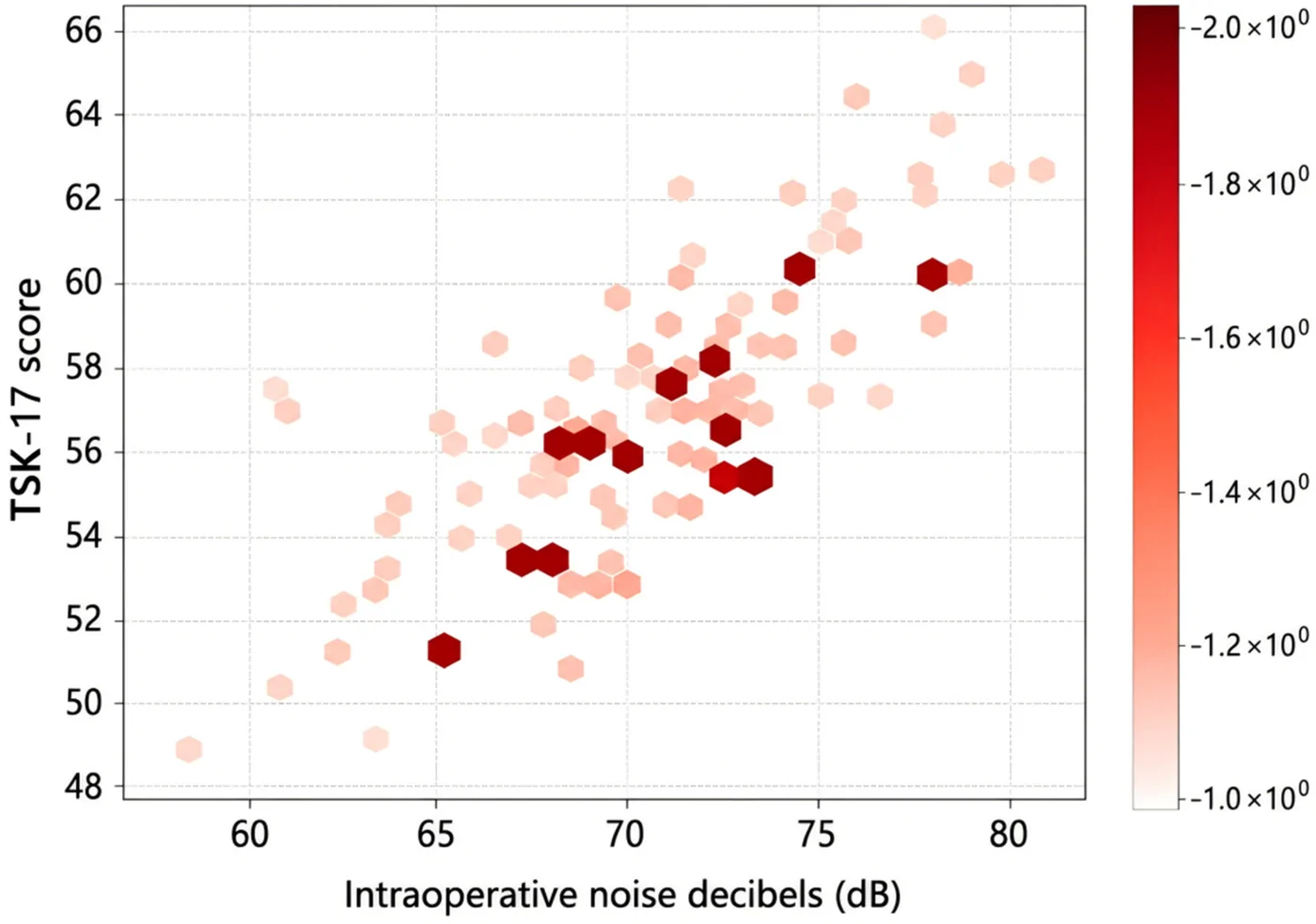
Heatmap showing the correlation between intraoperative noise levels in decibels and postoperative TSK-17 scores.

Intraoperative noise levels (decibels) showed significant positive correlations with TSK score (*r* = 0.6, *P* < 0.001) (Figure 2) and postoperative pain scores (VAS on day 3) (*r* = 0.594, *P* < 0.001), and a negative correlation with the HHS score (*r* = −0.471, *P* < 0.001).

**Figure 3:**
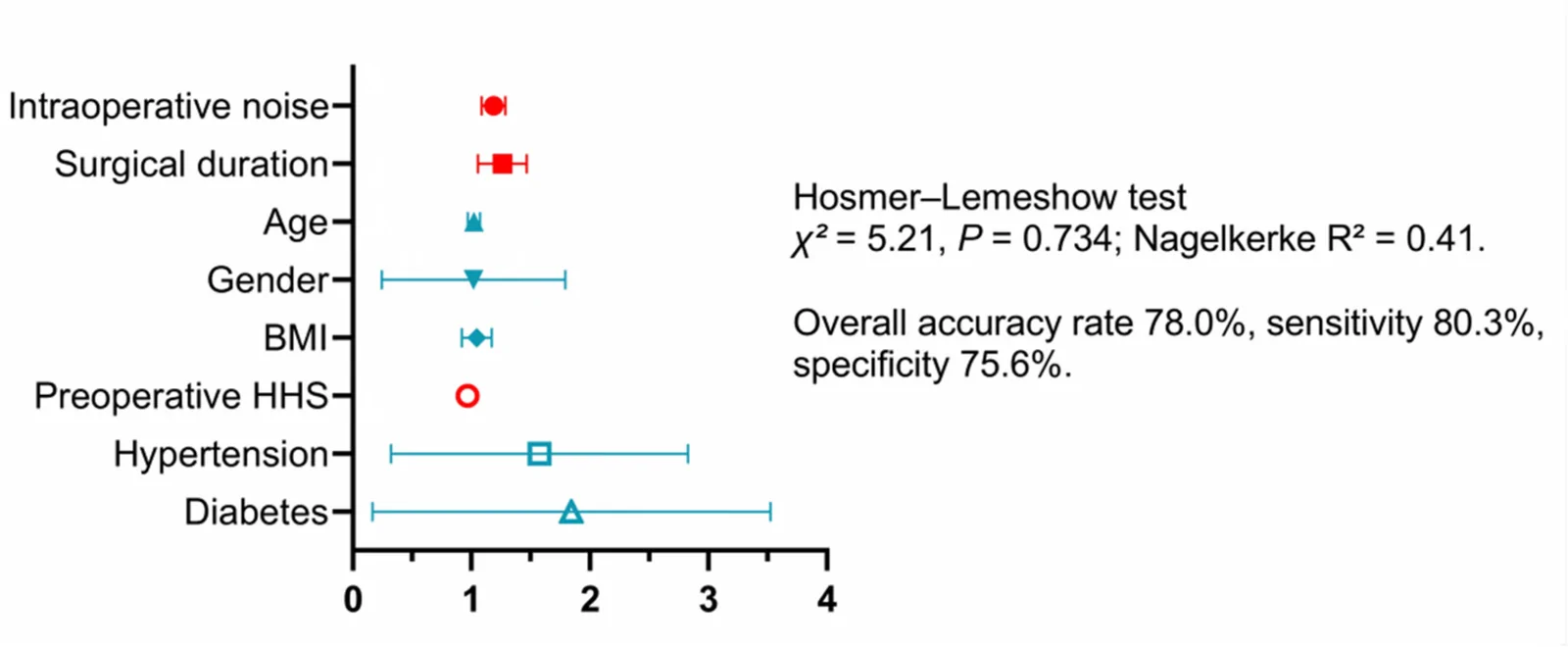
Forest plot of multivariate logistic regression identification of preoperative/intraoperative predictors of postoperative kinesiophobia.

A multivariate logistic regression prediction model was established using postoperative dyskinesia as the dependent variable (TSK ≥ 37 assigned a value of 1, TSK < 37 assigned a value of 0). The prediction model incorporated all available preoperative and intraoperative variables, including the intraoperative noise level (continuous variable), operative duration, age, sex, BMI, preoperative HHS score, and complications. Independent variables were selected using univariate regression analysis together with assessment of clinical significance, and were entered into the multivariate model using the Enter method. The results indicated that the intraoperative noise level was an independent predictor of postoperative kinesiophobia, with each 1 dB increase in noise level associated with an elevated risk (OR=1.18; 95% CI: 1.09–1.29; *P* < 0.001). Operative duration was also an independent predictor (OR=1.25 per 10 min; 95% CI: 1.06–1.47; *P* = 0.007), while the preoperative HHS score represented a protective factor (OR=0.97; 95% CI: 0.94–0.99; *P* = 0.012). The model exhibited good fit (Hosmer–Lemeshow test *P* = 0.734), with a Nagelkerke R² of 0.41, overall classification accuracy of 78.0%, sensitivity of 80.3% (Table 3), and specificity of 75.6% (Figure 3).

**Table 3:** Concurrent association model showing the association between kinesiophobia and variables on postoperative day 3.

Variables	*β*	OR	95% CI	*P*
Noise level > 70 dB	0.903	2.471	1.526–3.980	<0.001
Day 3 VAS score	0.564	1.758	1.321–2.340	<0.001
Day 3 HADS score	0.318	1.374	1.087–1.790	<0.001

OR, odds ratio; CI, confidence interval; VAS, visual analog scale; HADS, hospital anxiety and depression scale.

A concurrent association model was established to further elucidate the potential mechanisms underlying postoperative pain and anxiety. This model incorporated the VAS and HADS scores on postoperative day 3 (Table 4). This model was not intended to be predictive, and the results only confirm the strength of the independent association with postoperative kinesiophobi (Table 3).

**Table 4:** Multivariate logistic regression identification of preoperative/intraoperative predictors of postoperative kinesiophobia.

Variable	B	OR	95%Cl	P	VIF
Intraoperative noise (per 1 dB increase)	0.165	1.18	1.09–1.29	<0.001	1.62
Surgical duration (per 10 min increase)	0.223	1.25	1.06–1.47	0.007	1.58
Age (per 1 year increase)	0.018	1.02	0.97–1.07	0.438	1.10
Gender (male=1)	−0.198	0.82	0.36–1.87	0.635	1.06
BMI (per 1 kg/m?increase)	0.041	1.04	0.92–1.17	0.514	1.12
Preoperative HHS (per 1 point increase)	−0.031	0.97	0.94–0.99	0.012	1.18
Hypertension (yes=1)	0.214	1.24	0.52–2.96	0.625	1.08
Diabetes (yes=1)	0.287	1.33	0.48–3.72	0.583	1.07

OR, odds ratio; CI, confidence interval; VIF, variance inflation factor; BMI, body mass index; HHS, harris hip score; TSK, tampa scale for kinesiophobia.

## Discussion

The findings of this prospective cohort study indicated that patients exposed to higher levels of intraoperative noise during THA experienced significantly greater levels of postoperative pain, kinesiophobia, and functional impairment. Intraoperative noise was found to be an independent predictor of kinesiophobia (OR=1.18 per dB increase, 95% CI: 1.09–1.29, *P* < 0.001), with an AUC of 0.81, indicating satisfactory discriminative ability. These results suggest that intraoperative noise represents a modifiable environmental factor with potential clinical implications for perioperative risk stratification.

The association between intraoperative noise and postoperative kinesiophobia may involve multiple mechanisms. As outlined above, substantial evidence supports a link between surgical noise exposure and postoperative anxiety. First, Eelevated noise levels activate the sympathetic nervous system and the hypothalamic–pituitary–adrenal axis, inducing stress responses and negative emotional states that can amplify pain sensitivity (20–22). Pain-related anxiety and fear-avoidance beliefs represent critical contributors to postoperative kinesiophobia, and perceived stress has been reported to mediate the relationships between these psychological constructs and kinesiophobia. Accordingly, intraoperative noise may predispose patients to heightened kinesiophobia partially through stress- and anxiety-driven fear-avoidance pathways. Second, hHigh-intensity surgical noise may reinforce postoperative fear of movement and avoidance behavior (23). The reciprocal relationships among pain, fear, and functional impairment have been extensively documented, and the present findings suggest that intraoperative noise may represent an environmental trigger initiating this maladaptive cycle (24, 25).

Notably, the correlation between intraoperative noise and kinesiophobia scores (*r* = 0.60) exceeded the correlation between intraoperative noise and pain scores (*r* = 0.59), indicating that the noise–kinesiophobia relationship may extend beyond direct pain mediation (26). Possible mechanisms include auditory discomfort, elevated autonomic arousal, and the formation of intraoperative acoustic “traumatic memories,” consistent with previously proposed “sound stress–emotional generalization” (27, 28).

From a clinical perspective, the present results support the feasibility of monitoring intraoperative noise levels in real-time for perioperative risk stratification. The 70 dB threshold used in this study is consistent with the established comfort limits for healthcare environments (14, 20). Exceeding this threshold triggers autonomic stress and discomfort responses. The patients in the present cohort received spinal anesthesia and were thus essentially conscious during surgery, increasing the relevance of auditory stimuli (29). Exposure to sustained or sudden high-intensity noise from equipment such as drills and bone saws (for which peak noise levels often exceed 90 dB) may induce increased tension, discomfort, and a perceived lack of control, and may lead to negative expectations regarding postoperative movement and pain (14). Given these psychological burdens caused by surgical noise, perioperative music or other positive auditory stimuli, together with intraoperative noise isolation via noise-cancelling headphones, can mitigate the adverse psychological outcomes induced by high-decibel intraoperative noise. Further mitigation strategies include optimising surgical instruments and operating-room environmental conditions, such as adopting ultrasonic bone scalpels to lower peak procedural noise and incorporating sound-attenuating materials into operating-room architectural design.

Consistent with previous studies linking factors associated with surgical environment to postoperative outcomes, the present findings contribute to an emerging body of evidence indicating the importance of non-pharmacological, environmental influences on patient recovery (30). While previous research has demonstrated associations between kinesiophobia and preoperative psychological factors and pain catastrophizing, the role of intraoperative auditory stimuli has received limited attention (9, 26). This study addresses this gap by demonstrating a robust and independent effect of intraoperative noise on postoperative kinesiophobia (23).

This study used a multivariate logistic regression model incorporating available preoperative and intraoperative variables. The results indicated that the level of intraoperative noise was independently predictive of the development of postoperative kinesiophobia, with each 1 dB increase in noise level correlating with an elevated risk of dyskinesia (OR=1.18; 95% CI: 1.09–1.29; *P* < 0.001). These findings suggest the feasibility of using real-time intraoperative noise monitoring for perioperative risk stratification. ROC curves indicated that intraoperative noise exhibited strong discriminative power for kinesiophobia (AUC = 0.814), achieving high sensitivity and specificity at 72.3 dB and thereby supporting the use of intraoperative environmental monitoring for identifying high-risk populations for postoperative kinesiophobia. ROC curves showed intraoperative noise predicted kinesiophobia with an AUC = 0.814; optimal cutoff = 72.3 dB (sensitivity = 82.1%, specificity = 75.4%). Lastly, VAS and HADS scores on postoperative day 3 were both independently associated with kinesiophobia (both *P* < 0.01). Additionally, the concurrent association model indicated that pain and anxiety levels on postoperative day 3 were independently associated with kinesiophobia. This finding demonstrates the influence of noise on motor avoidance behavior via pain perception and emotional responses, although this does not form a basis for the predictive model (31). In this study, patients received 37.44 ± 5.12 μg of sufentanil during surgery, and the number of effective presses on the PCA in the first 72 h after surgery was 4.3 ± 2.8 times. These numbers are basically in line with the dosage range reported in previous studies for hip replacement under general anesthesia (32).

This study focuses on kinesiophobia as a critical psychological outcome in rehabilitation and provides evidence for the clinical importance of noise management (33, 34). The key strengths of this study include its prospective design, standardized outcome assessment using validated instruments (TSK, VAS, HHS), and comprehensive statistical evaluation. The identification of intraoperative noise as an independent predictor of kinesiophobia provides a novel target for perioperative interventions. Nevertheless, several limitations should be acknowledged: the 70 dB cutoff value warrants further validation; The use of equivalent continuous sound level throughout the procedure in this study may lead to an underestimation of exposure to impulsive noise. Future studies should use peak noise accumulation parameters.the single-center. design may limit generalizability of the findings; longer-term follow-up is needed; and residual confounding cannot be excluded despite statistical adjustment. Future research should validate the present findings in larger, multicenter cohorts and explore the efficacy of using noise-reduction interventions (e.g., noise-absorbing materials, quieter surgical equipment, and music therapy) in mitigating the development of postoperative kinesiophobia. Examination of the mediating roles of stress hormones, autonomic indicators, and psychological factors would further elucidate the underlying mechanisms. Additionally, the development of clinical prediction models integrating intraoperative noise with other risk factors may enhance personalized perioperative care.

In conclusion, the level of intraoperative noise is an independent predictor of the development of postoperative kinesiophobia in patients undergoing THA, with higher noise levels associated with increased risk. These findings support the potential utility of monitoring the levels of intraoperative noise for perioperative risk stratification and highlight the importance of addressing environmental factors in surgical care.

## Limitation

1. This retrospective may underestimate impulsive noise exposure and calling for future prospective studies to adopt peak noise accumulation parameters. 2. Future studies should use peak noise accumulation parameters. 3. Furthermore, we failed to perform paired comparison of preoperative and postoperative TSK-17 scores within different noise exposure groups due to the lack of complete paired preoperative kinesiophobia data. Although baseline TSK-17 values were adjusted in the regression model to control for confounding effects, we could not quantify intra-individual changes in kinesiophobia attributable to intraoperative noise exposure. Future studies with complete paired perioperative psychological assessments are needed to validate and quantify the influence of intraoperative noise on postoperative kinesiophobia changes.

## Data Availability

The original contributions presented in the study are included in the article/Supplementary Material, further inquiries can be directed to the corresponding author.
